# Decoding Health: Exploring Essential Biomarkers Linked to Metabolic Dysfunction-Associated Steatohepatitis and Type 2 Diabetes Mellitus

**DOI:** 10.3390/biomedicines13020359

**Published:** 2025-02-04

**Authors:** Sulagna Mukherjee, Seung-Soon Im

**Affiliations:** Department of Physiology, Keimyung University School of Medicine, Daegu 42601, Republic of Korea

**Keywords:** biomarkers, obesity, MASH, type 2 diabetes, metabolic disorder

## Abstract

The investigation of biomarkers for metabolic diseases such as type 2 diabetes mellitus (T2DM) and metabolic dysfunction-associated steatohepatitis (MASH) reveals their potential for advancing disease treatment and addressing their notable overlap. The connection between MASH, obesity, and T2DM highlights the need for an integrative management approach addressing mechanisms like insulin resistance and chronic inflammation. Obesity contributes significantly to the development of MASH through lipid dysregulation, insulin resistance, and chronic inflammation. Selective biomarker targeting offers a valuable strategy for detecting these comorbidities. Biomarkers such as CRP, IL-6, and TNF-α serve as indicators of inflammation, while HOMA-IR, fasting insulin, and HbA1c are essential for evaluating insulin resistance. Additionally, triglycerides, LDL, and HDL are crucial for comprehending lipid dysregulation. Despite the growing importance of digital biomarkers, challenges in research methodologies and sample variability persist, necessitating further studies to validate diagnostic tools and improve health interventions. Future opportunities include developing non-invasive biomarker panels, using multiomics, and using machine learning to enhance prognoses for diagnostic accuracy and therapeutic outcomes.

## 1. Introduction

In recent years, the exploration of biomarkers, particularly in metabolic dysfunction-associated steatohepatitis (MASH) and type 2 diabetes mellitus (T2DM), has garnered considerable attention. Numerous comprehensive analyses have highlighted the significant overlap of metabolites and pathways associated with obesity and diabetes, elucidating the potential of biomarkers in unraveling these complex diseases. The strong association between MASH, obesity, and T2DM emphasizes the need for an integrative approach to managing these interconnected metabolic disorders, focusing on several overlapping mechanisms such as insulin resistance, chronic inflammation, lipid dysregulation, and adipokine imbalance. Among the globally prevalent metabolic disorders, obesity and T2DM are estimated to impact over 8.5% of adults worldwide, while the prevalence of metabolic dysfunction-associated liver disease (MASLD) and MASH in patients with T2DM is almost 55.5% to 37.3% [1,2].

In general, excess adipose tissue contributes to the abnormal accumulation of lipids within the liver, establishing obesity as a well-recognized risk factor for MASH development [3]. Elevated levels of free fatty acids and inflammatory cytokines released from visceral fat can lead to hepatocellular injury, triggering the inflammatory cascade that is characteristic of MASH [4]. This hepatic steatosis is often exacerbated by insulin resistance, a hallmark of metabolic dysfunction that not only predisposes individuals to obesity and T2DM but also accelerates the progression of steatosis into more severe liver injury [5]. Typically, this cluster of disorders is collectively termed metabolic syndrome, which requires the presence of the following diagnostic criteria: abdominal obesity with higher waist circumference, high triglyceride content (≥150 mg/dL), low high-density lipoprotein (HDL) cholesterol (<40 mg/dL (men) or <50 mg/dL (women)), high blood pressure (≥130/85 mm Hg), and high fasting glucose (≥100 mg/dL) [6]. Although all metabolic disorders pose serious health threats, the progression of MASH significantly elevates the risk of liver cancer, with liver-related mortality increasing more than 10-fold [1]. T2DM further complicates this relationship, as hyperglycemia and altered insulin signaling perpetuate a cycle of metabolic dysfunction. Individuals with T2DM frequently display elevated liver enzymes, indicative of hepatic stress and injury, and studies have shown that the prevalence of liver steatosis and MASH is markedly higher in populations with diabetes than in people without diabetics [7]. The pathophysiological link between insulin resistance and hepatic steatosis is particularly robust, as insulin not only regulates glucose metabolism but also plays a vital role in lipid synthesis and degradation. Clinically, MASH is associated with changes in various circulating markers of inflammation and fibrosis, including extracellular matrix components [8].

Biomarkers provide critical insights into the presence and progression of these conditions. Defined as measurable parameters reflecting specific features of disease-related pathophysiological processes, biomarkers have gained prominence in the field of metabolic disorders [9]. Over the past decade, the number of digital biomarker studies indexed in PubMed has increased by 25%, indicating the growing importance of this field [10]. Moreover, biomarkers have the potential to provide valuable biomedical insights and improve decision-making in healthcare across a wide range of diseases, from movement-related disorders and breast cancer to Alzheimer’s disease [9]. In context of interconnected mechanisms in metabolic disorders, many key biomarkers have gained attention, including homeostatic model assessment for insulin resistance (HOMA-IR), fasting insulin, glycated hemoglobin (HbA1c), C-reactive protein (CRP), Interleukin-6 (IL-6), Tumor Necrosis Factor-alpha (TNF-α), total cholesterol (low-density lipoprotein (LDL) and HDL), and triglycerides (TGs) (Table 1).

However, it is crucial to acknowledge the limitations inherent in the current research landscape. Variability in analytical methods and sample characteristics can pose challenges, potentially impeding the identification of reliable biomarkers. Nonetheless, this review highlights pivotal studies that illuminate the intricate relationships between metabolic alterations and associated biomarkers, emphasizing the necessity for further investigations. Such efforts could validate definitive diagnostic tools while simultaneously paving the way for more targeted and effective interventions to address prevalent health issues.

## 2. Biomarkers in Insulin Resistance

Insulin resistance is characterized by a diminished response of target tissues to insulin, leading to impaired glucose homeostasis and subsequent metabolic derangements. The identification and understanding of reliable biomarkers are essential for early diagnosis, risk stratification, and the development of targeted therapeutic interventions [11]. Various biomarkers serve as tools for assessing insulin sensitivity and resistance, with HOMA-IR, fasting insulin levels, and HbA1c being particularly prominent. These biomarkers not only provide valuable insights into an individual’s glucose metabolism but also aid in the early detection and management of insulin-related disorders. As researchers continue to investigate the significance of these indicators, it is imperative to examine their relative strengths, limitations, and clinical applications. The following subsections delve into the roles and effectiveness of HOMA-IR, fasting insulin, and HbA1c in evaluating insulin resistance, ultimately highlighting their importance in guiding therapeutic interventions and improving outcomes for patients at risk for metabolic complications.

### 2.1. HOMA-IR as a Biomarker

HOMA-IR is a prominent tool for assessing insulin resistance in clinical diagnosis. Nonetheless, there is significant variation in the HOMA-IR threshold levels used to determine insulin resistance [12]. Various geographical regions have conducted population-based studies to establish cut-off values of HOMA-IR for diagnosing insulin resistance. In a study involving 12,313 participants, it was found that the optimal HOMA-IR cut-off for identifying metabolic syndrome was 2.35, with variations noted in BMI categories [13]. Moreover, another study indicated the threshold for HOMA-IR being considered pathological to be 2.5 in an adult Caucasian population, whereas for young Caucasians, the threshold might be higher [14,15]. This highlights the importance of using tailored HOMA-IR thresholds to diagnose insulin resistance and metabolic syndrome in diverse populations [13,14,15]. Emerging research suggests that traditional measures like HOMA-IR may lack sensitivity when compared to newer biomarkers such as circulating microRNAs, which have been proposed as potential predictors of diabetes [16]. HOMA-IR effectively bridges this gap by offering a straightforward calculation that can enhance the early detection of insulin resistance, which is crucial for preventing the progression of type 2 diabetes. While HOMA-IR is often used in conjunction with other biomarkers like fasting insulin and HbA1c, the integration of novel markers such as microRNAs is reshaping the biomarker landscape [17]. HOMA-IR serves as a widely used metric to estimate insulin sensitivity from fasting glucose and insulin levels and is calculated using the following formula: HOMA-IR = (fasting insulin (μU/mL) × fasting glucose (mg/dL))/405. This index provides a simple and cost-effective means of assessing insulin resistance without requiring complex testing. Clinically, elevated HOMA-IR values indicate an impaired insulin response, suggesting a predisposition to metabolic disorders such as type 2 diabetes and cardiovascular disease. In a cohort of high-risk youth with obesity, one study illustrated significant correlations between insulin resistance and elevated fasting insulin levels, further emphasizing the clinical relevance of HOMA-IR in identifying patients at risk for worsening metabolic dysfunction [18]. Thus, HOMA-IR is invaluable not only for diagnosis but also for informing treatment strategies aimed at reducing insulin resistance and improving metabolic health.

### 2.2. Fasting Insulin Levels

Elevated fasting insulin is a key indicator of insulin resistance, a condition in which cells become less responsive to insulin. This condition not only suggests potential prediabetes but also raises the risk of developing type 2 diabetes. A study on women with gestational diabetes revealed significant increases in fasting insulin and related factors, highlighting the relationship between elevated insulin levels and insulin resistance [19]. For instance, recent research indicates that alongside traditional markers like HbA1c, the combination of specific circulating microRNAs may enhance predictive accuracy for diabetes onset [16]. In clinical practice, measuring fasting insulin levels provides a straightforward and comprehensive picture of an individual’s metabolic state. When interpreted in comparison with other biomarkers, such as HOMA-IR, fasting insulin offers a more nuanced understanding of metabolic health [20]. A rise in fasting insulin often correlates with heightened insulin resistance, reflecting the pancreas’s compensatory efforts to produce more insulin to maintain normal glucose levels. This is supported by studies linking inflammatory biomarkers, such as high-sensitivity C-reactive protein, to changes in insulin dynamics, which exacerbate insulin resistance and contribute to the pathology of diabetes [9]. Conversely, lower fasting insulin levels suggest improved insulin sensitivity, which is critical for maintaining metabolic homeostasis [20]. Thus, tracking fasting insulin levels is essential for early intervention and prevention strategies in at-risk populations. Additionally, incorporating fasting insulin measurements into clinical assessments provides valuable insights for predicting and managing conditions related to insulin resistance.

### 2.3. HbA1c as a Biomarker

HbA1c is a pivotal biomarker in the diagnosis and management of T2DM. HbA1c is formed through a non-enzymatic reaction between glucose and hemoglobin. The concentration of HbA1c in the blood is proportional to the average glucose level; thus, higher levels of HbA1c indicate poorer glycemic control [21]. Based on the results of a previous study, the period over which glycemic control was reflected in a comparison of good and poor glycemic controls was related to the duration of diabetes, which was observed for approximately 2.5 years in both males and females. According to the American Diabetes Association (ADA), an HbA1c level of 6.5% or higher indicates diabetes, while levels between 5.7% and 6.4% denote prediabetes [22]. This classification enables healthcare professionals to stratify risk and implement early interventions. The advantages of employing HbA1c as a biomarker are manifold. First, its objective nature allows for standardized testing, which enhances the reliability of diabetes diagnosis across diverse populations. Furthermore, HbA1c is less influenced by daily fluctuations in blood glucose levels than traditional self-monitoring methods, providing a more stable measure of glycemic control [23]. Additionally, it is worth noting that HbA1c testing is widely accessible and can be performed in various healthcare settings, making it a practical choice for routine assessments. However, it is essential to acknowledge the limitations inherent in the use of HbA1c as a biomarker. Conditions affecting erythrocyte turnover, such as hemolytic anemia or recent blood transfusions, can distort HbA1c results [24]. Moreover, variations in hemoglobin variants (e.g., hemoglobin S or C) may also lead to inaccuracies in specific populations. It is crucial to recognize that HbA1c levels can be influenced by factors other than blood glucose, such as alterations in the penetration of glucose through erythrocyte membranes, the rate of glycosylation, and the lifespan of erythrocytes [25]. The factors which result in falsely elevated HbA1c levels are anemias associated with decreased red cell turnover due to iron deficiency, Vitamin B-12 deficiency, and folate deficiency [26]. Other reasons include uremia, severe hypertriglyceridemia (when the level >1750 mg/dL), severe hyperbilirubinemia (when the level >20 mg/dL), and chronic alcohol consumption resulting in the formation of an acetaldehyde–HbA1 compound [26]. Alternatively, there are factors leading to falsely low Hb1Ac levels: for instance, liver cirrhosis [27], hyperbilirubinemia [28], and conditions that reduce red blood cell formation [26]. Therefore, while HbA1c remains an invaluable tool for diabetes management, it should be interpreted alongside other clinical parameters and patient histories to ensure an accurate assessment of glycemic status [22,29]. Ongoing research continues to refine the application of HbA1c, further enhancing its clinical utility.

## 3. Biomarkers in Chronic Inflammation

Insulin resistance often triggers a state of low-grade chronic inflammation, activating immune cells and releasing pro-inflammatory cytokines like CRP, IL-6, and TNF-α. This inflammation exacerbates liver damage in MASH, leading to steatohepatitis, and worsens insulin resistance in T2D [30].

### 3.1. CRP as a Biomarker

CRP is an acute-phase reactant synthesized by the liver in response to inflammatory stimuli. Its levels in the blood rise rapidly during acute inflammation and can serve as a sensitive marker for detecting systemic inflammation [31]. CRP belongs to the pentraxin (PTX) family of proteins with a short-chain structure and is synthesized by hepatocytes in response to inflammatory signals [32]. The production of CRP is typically induced by the combination of IL-6 and IL-1β, triggering an acute-phase response. Its promoter region includes binding sites for the liver-specific transcription factor, hepatocyte nuclear factor (HNF1), along with two binding sites for C/EBPβ (CCAAT/enhancer-binding protein β), which are associated with IL-6-induced transcription [33]. Transcription factors, including STAT3, Rel p50, c-Rel, and C/EBPβ/δ, predominantly regulate CRP expression. STAT3 and Rel bind near the CRP promoter within the non-coding region, facilitating the proximity of C/EBP to the nucleic acid, thereby enhancing CRP expression [34].

Although CRP is generally linked with acute inflammation, higher levels can remain present in chronic inflammatory conditions, suggesting continuous tissue damage and immune system activation. Recent clinical research has shown a connection between elevated CRP levels and the prevalence of T2DM [35]. Examining CRP levels in conjunction with conventional metabolic markers can provide a more comprehensive understanding of the inflammatory pathways influencing the progression of T2DM. This insight could guide the development of improved prevention and management strategies.

### 3.2. IL-6 as a Biomarker

IL-6 is a multifunctional cytokine implicated in diverse biological processes, including inflammation, immune response, and hematopoiesis. It plays a pivotal role in the acute-phase response by inducing the synthesis of CRP and other acute-phase proteins [36]. Even though the expression of IL-6 is controlled through several pathways, the primary triggers for IL-6 expression are TNF-α and IL-1β, which are two key upstream pro-inflammatory cytokines. Additionally, other cytokines, adipokines, prostaglandins, and Toll-like receptors also stimulate IL-6 production [37]. Several studies have shown that individuals with T2DM have higher levels of IL-6 and CRP, likely due to an excess of adipose tissue [38,39]. Interestingly, the IL-6 levels of people with obesity, including those without T2DM, are therefore higher. The overall catabolic effect of the physiological levels of IL-6 in adipose tissue is probably the reason why anti-IL-6 medication is linked to an increase in body weight. Additionally, the overproduction of IL-6 in adipose tissue macrophages may worsen insulin resistance and encourage hepatic gluconeogenesis. People with obesity, including those without T2D, have higher IL-6 levels [37,40]. Earlier studies demonstrate that IL-6 influences CRP generation and is associated with both liver and adipose inflammation, contributing to MASH development. TNF-α influences the effects of IL-6 by acting as an upstream mediator. Previous research indicates that extreme obesity has been shown to elevate CRP levels in the liver and adipose tissues, with little distinction between fatty liver and MASH. Essentially, there is a positive correlation between the amounts of CRP mRNA in these tissues. These results imply a role for IL-6 in the progression of MASH. An earlier report stated that IL-6 signaling played a protective role against the progression of hepatic steatosis; however, this could result in enhanced liver inflammation. Using anti-IL6 as a therapeutic target would require the proper optimization of the dose and administration due to the lack of any direct evidence of using anti-IL6 as a therapy in MASH models, although studies in related conditions suggest potential benefits [41,42]. Furthermore, new studies show a robust relationship between systemic insulin resistance, fibrosis stage, hepatic inflammation severity, hepatocyte IL-6 expression, and plasma IL-6 levels [43].

### 3.3. TNF-α as a Biomarker

TNF-α is a potent pro-inflammatory cytokine that plays a central role in the pathogenesis of numerous inflammatory diseases. It is produced by various cell types, including macrophages, T cells, and endothelial cells, and exerts its effects through a complex network of signaling pathways [44]. Studies have shown that people with metabolic syndrome and T2DM frequently have higher levels of TNF-α, indicating a connection between prolonged inflammation and metabolic dysregulation. Elevated TNF-α concentrations might play a role in the inflammatory environment that worsens insulin resistance and disrupts glucose metabolism [45,46]. Moreover, the interaction between TNF-α and other inflammatory mediators can establish a feedback loop that continues the cycle of inflammation and metabolic dysfunction, underscoring the need to monitor TNF-α levels in these patients [47]. Investigating TNF-α levels in the context of MASH and T2DM enhances our understanding of the underlying mechanisms of these conditions while revealing potential therapeutic targets. By targeting TNF-α with pharmacological drugs or lifestyle changes, we may find an effective way to alleviate the inflammatory processes contributing to insulin resistance and metabolic syndrome [48]. As clinical research continues to shed light on the role of TNF-α in these metabolic disorders, it becomes evident that addressing inflammation might be crucial for managing and preventing T2DM and associated conditions.

## 4. Biomarkers of Lipid Dysregulation

Lipid imbalance, marked by atypical lipid levels in the bloodstream, poses a major risk for various diseases, such as cardiovascular disease, metabolic syndrome, obesity, and T2DM. Tracking lipid biomarkers is crucial for the early identification and management of lipid imbalance and cardiovascular disorders. Understanding the fundamental processes of lipid metabolism and recognizing high-risk individuals allows clinicians to implement preventive strategies and initiate appropriate treatment plans, including lifestyle changes, pharmacological therapy, or a combination of both [49,50]. A variety of biomarkers are used to assess lipid dysregulation, providing valuable insights into the underlying pathophysiology.

### 4.1. Total Cholesterol as a Biomarker

Total cholesterol is an overall measurement of cholesterol within the bloodstream, including LDL, HDL, and triglycerides. Elevated total cholesterol levels, particularly a high LDL and low HDL, are well-known risk factors for coronary heart diseases [51]. Increased cholesterol levels can exacerbate liver inflammation and scarring due to fat accumulation in the liver, contributing to the development of MASH [52]. In individuals with T2DM, the interaction between insulin resistance and lipid metabolism typically results in dyslipidemia, marked by increased total cholesterol and triglycerides. This condition increases the risk of atherosclerosis, emphasizing the importance of cholesterol management in diabetes care. By monitoring and improving cholesterol levels through lifestyle interventions and medication, practitioners can mitigate the risks linked with these metabolic disorders [53].

Atherogenic dyslipidemia, characterized by elevated levels of small dense LDL and reduced levels of HDL, is not consistently observed in individuals with MASLD. However, dyslipidemia becomes an independent risk factor for liver fibrosis only when MASLD is combined with T2DM, not in MASLD alone [54]. LDL cholesterol, commonly known as “bad” cholesterol, plays a role in the formation of atherosclerotic plaques. Various population-based studies have consistently shown that elevated LDL levels significantly contribute to cardiovascular mortality and T2DM [55]. On the other hand, HDL, commonly known as the “good” cholesterol, aids in the removal of excess cholesterol, reducing the risk of heart disease. Low HDL has been identified as a factor related to advanced fibrosis in T2DM [56]. HDL facilitates the reverse transport of cholesterol from peripheral tissues, including the arterial wall, back to the liver. Additionally, HDL displays properties such as anti-inflammatory, antioxidant, and anti-thrombotic effects, alongside the restoration of endothelial function, functions partly enabled by its enrichment with apoA1 or enzymes [57]. Patients with T2D experience significant alterations in cholesterol metabolism, marked by lowered plasma concentrations of campesterol, which serves as an indicator of cholesterol absorption, and heightened plasma levels of lathosterol, indicative of cholesterol synthesis. Furthermore, a correlation has been noted between increased liver fat and elevated plasma lathosterol levels. This correlation may be due to the increased activity of SREBP2, an important regulator of cholesterol uptake and synthesis. These changes illustrate a reduction in cholesterol absorption along with a rise in cholesterol synthesis in these patients [58]. In addition to these traditional lipid biomarkers, other emerging biomarkers that provide additional insights into the pathophysiology of lipid dysregulation and cardiovascular disease risk have also been identified, such as lipoprotein A, apolipoprotein B, and cholesterol ester transfer protein.

### 4.2. Triglycerides as a Biomarker

Triglycerides are the predominant form of fat present in the body and foods. Stored in fat cells, they serve as an essential energy source. While triglycerides play a vital role in numerous bodily functions, elevated levels can lead to significant health issues, including heart disease, stroke, and diabetes [56]. Extended lipid exposure, as seen in obesity or metabolic syndrome, can result in triglyceride accumulation in beta cells, impairing their cellular functions, inducing metabolic stress. This condition, termed lipotoxicity, triggers inflammatory pathways and the release of pro-inflammatory cytokines, which can lead to beta-cell dysfunction or death. The subsequent metabolic and immune responses from beta-cell dysfunction and high blood sugar can activate inflammatory pathways, causing hepatocyte damage and progression from steatosis to MASH [59]. In individuals with T2DM, triglyceride levels are elevated mainly because of two reasons: due to the excessive production of triglyceride-rich very-low-density lipoprotein (VLDL1) by the liver and the sluggish removal of triglyceride-rich lipoproteins (TRLs). This excessive production is associated with the disruption of liver lipid metabolism, involving an increase in de novo lipogenesis (DNL) and the reduced suppression of VLDL1 release by insulin. The slow removal of these lipoproteins is due to diminished lipoprotein lipase (LPL) activity and a decrease in the liver’s uptake of TRL remnants [60].

### 4.3. ALT and AST as Biomarkers

ALT and AST are key liver enzymes most commonly used to detect liver damage. Clinical practice guidelines recommend screening individuals with T2DM for advanced liver fibrosis and MASH, a process often facilitated by measuring these two biomarkers [61]. Despite their utility as conventional biomarkers, ALT and AST levels in serum typically range between 7 and 55 units per liter (U/L) for ALT and 8 to 49 (U/L) for AST. The AST:ALT ratio should normally be less than one, but in 92% of patients with alcoholic liver disease, it is greater than one, and in 70% of cases, it is greater than two. Therefore, AST:ALT levels more than two imply alcoholic liver disease, whereas scores less than one are indicative of NAFLD or NASH [62]. However, despite their widespread use, ALT and AST lack the sensitivity and specificity necessary for the early identification of and separation between MASLD and MASH. This limitation poses a challenge in leveraging these traditional biomarkers for more diagnostic and therapeutic purposes [63].

## 5. miRNAs as Biomarkers

MicroRNAs (miRNAs) are small non-coding RNAs that play crucial roles in regulating gene expression and have emerged as potential biomarkers for various diseases, including T2DM and MASH. Irrespective of their origin, miRNAs act as transcription controllers by suppressing mRNA with assistance from the RNA-induced post-transcriptional gene silencing complex, and they can also associate with gene-promoting areas to enhance gene transcription. When miRNAs are released into the bloodstream and carried in vesicles, they are able to reach different tissues and cells. They enable cellular communication, leading to a synchronized reaction among different cell types without changing the DNA structure, which is a form of epigenetic regulation. As a result, the miRNA composition in blood plasma indicates a person’s health condition, regardless of whether they are healthy or have a disease [64]. Certain biomarkers are only evident in obesity, for instance, mir-15a, mir-365, mir-375, and mir-222 [65]. However, several miRNAs have been identified as potential biomarkers for both MASH and T2DM. These common miRNA biomarkers include miR-122, miR-126, miR-144, miR-29a, miR-203, and miR-223. In the case of miR-122 and miR-126, decreased levels have been reported in T2DM and are also associated with cardiovascular complications, which can occur in both MASH and T2DM [16,64,66]. miR-144 has been identified as a potential biomarker for insulin resistance, whereas the dysregulation of miR-29a has been observed in both individuals with prediabetes and individuals with diabetes, and it may also play a role in liver fibrosis associated with MASH [64,67]. miR-103 has been linked to adipose tissue regulation and glucose metabolism, which are relevant to both MASH and T2DM, and altered levels of miR-223 have been reported in patients with T2DM and may also be involved in liver inflammation associated with MASH [16,68]. These miRNAs appear to hold potential as biomarkers for both MASH and T2DM, as they highlight the common metabolic disturbances associated with these conditions. Nonetheless, additional studies are necessary to confirm their clinical relevance and distinctiveness for each disease.

## 6. Future Prospects for the Utilization of These Biomarkers

**Figure 1:**
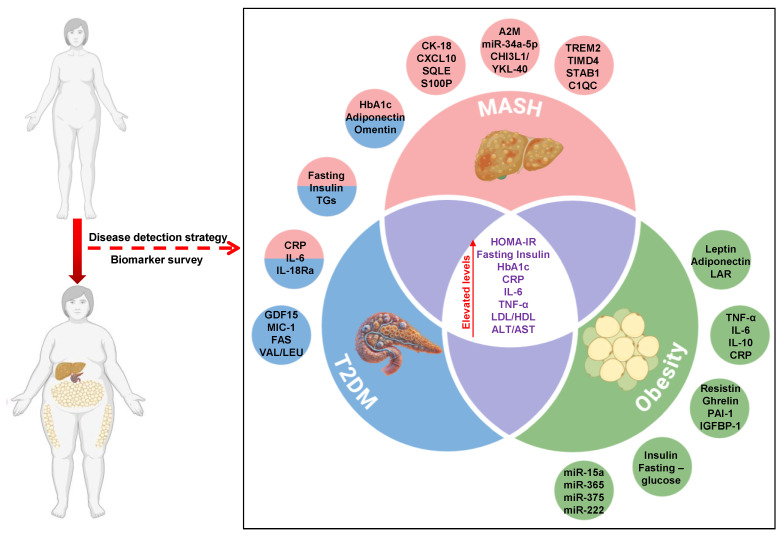
Overlapping biomarkers are responsible for metabolic disorders. This Venn diagram represents the common biomarkers used to diagnose MASH, T2D, and obesity in humans. Created with BioRender.com.

An effective method to manage patients with T2DM and MASH involves the strategic use of overlapping biomarkers (Figure 1). Through the development of non-invasive biomarker panels for early diagnosis, it is possible to achieve risk stratification. The objective is to identify the early stages of MASH and T2DM. Employing ’multiomics’ techniques, which integrate data from various biological sources (such as genomics and proteomics), and customizing both pharmacological and lifestyle interventions based on each patient’s specific biomarker profiles as personalized treatments would be highly advantageous. Another strategy could involve creating predictive models through machine learning to enhance the accuracy of predictions regarding liver fibrosis and other adverse effects. Emphasizing the use of overlapping biomarkers would generally boost prediction accuracy. Moreover, combining biomarkers with ’exposome’ data, which encompass the entirety of an individual’s environmental exposures over their lifetime, could provide more effective insights into the role of environmental factors in the progression of T2DM and MASH. Through the utilization of overlapping biomarkers and integration of diverse data sources, prediction accuracy and therapeutic precision can be improved.

## 7. Conclusions

Overall, this study highlights the critical role of overlapping biomarkers in various pathophysiological mechanisms, emphasizing their potential in an integrated and personalized approach to diagnosing, treating, and managing MASH in patients with T2DM. Leveraging advanced technologies and holistic data integration represents a promising pathway for achieving better diagnostic accuracy and therapeutic outcomes.

## Figures and Tables

**Table 1:** Key biomarkers influencing both MASH and T2D through overlapping mechanisms.

Biomarker	Mechanism	Tissue of Origin/Synthesis	Normal Level	Level in Disease State	Role in MASH	Role in T2D	Limitations
HOMA-IR	Insulin Resistance	Derived from glucose and insulin levels	<2.5	Elevated (>2.9)	Indicates hepatic insulin resistance; associated with fat accumulation, liver dysfunction	Indicates systemic insulin resistance; correlates with glucose dysregulation and beta-cell dysfunction	Lack of universal cut-off due to age-related differences in MASH; Reduced accuracy in lean patients with T2D; Compromised pancreatic function.
Fasting Insulin	Insulin Resistance	Pancreatic beta cells	2–25 µIU/mL	Elevated in MASH and early T2D	Increased due to hepatic insulin resistance, contributing to steatosis and inflammation	Elevated in early stages; declines with beta-cell failure in advanced stages	Reduced accuracy with abnormal glucose levels; Limited utility in insulin-treated patients; Paradoxical results in diabetic patients, due to increase in glucose-to-insulin (G/I) ratio; Lack of standardization.
HbA1c	Insulin Resistance	Circulating blood (glycation of hemoglobin)	<5.7%	Elevated (>6.5%)	Indicates chronic hyperglycemia; correlates with liver damage severity	Reflects long-term glycemic control; higher levels worsen complications	Inability to detect glucose variability; Reduced accuracy in glycemic control in patients with abnormal hemoglobin, altered red blood cell lifespan, or chronic kidney disease; Limited utility in elderly patients; Inadequate reflection of insulin resistance.
CRP	Chronic Inflammation	Liver (hepatocytes)	<1 mg/L	Elevated (>3 mg/L)	Elevated due to systemic and hepatic inflammation; a marker of liver fibrosis	Elevated in systemic inflammation and cardiovascular risks	Variability across populations; Limited predictive value; Lack of specificity; Limited utility in treatment monitoring.
IL-6	Chronic Inflammation	Adipose tissue, macrophages	<4 pg/mL	Elevated in MASH and T2D	Promotes hepatic inflammation and fibrosis	Contributes to systemic inflammation, insulin resistance, and beta-cell dysfunction	Limited predictive value; Lack of specificity for MASH; Variability in levels due to complex glucose metabolism in T2D.
TNF-alpha	Chronic Inflammation	Macrophages, adipose tissue	<2 pg/mL	Elevated in MASH and T2D	Triggers hepatic insulin resistance and apoptosis, promoting fibrosis	Impairs insulin signaling, contributing to systemic insulin resistance	Similar to IL-6: Limited predictive value; Lack of specificity for MASH; Variability in levels due to complex glucose metabolism in T2D.
TG (Triglyceride)	Lipid Dysregulation	Liver, adipose tissue, intestine, chylomicrons	<150 mg/dL	Elevated (>150 mg/dL)	Elevated due to hepatic lipid accumulation and reduced clearance	Elevated, contributing to atherogenic dyslipidemia	Lack of specificity; Influence of medications affect lipid metabolism, altering T levels; Indirect measure of insulin resistance.
LDL (Low-Density Lipoprotein)	Lipid Dysregulation	Liver	<100 mg/dL	Reduced in MASH, variable in T2D	Reduced in MASH due to altered lipid metabolism	Increased small, dense LDL, increasing cardiovascular risk	Reduced accuracy at low levels; Less reliable when triglyceride levels are high in T2D; Variability in calculation methods.
ALT (Alanine Amino- transferase)	Lipid Dysregulation	Liver	<40 U/L	Elevated (>40 U/L)	Marker of hepatocyte damage and inflammation	Elevated levels with fatty liver	Variability in cut-off values; Poor correlation with disease severity.
AST (Aspartate Amino- transferase)	Lipid Dysregulation	Liver	<35 U/L	Elevated (>35 U/L)	Reflects liver injury and mitochondrial dysfunction	Elevated levels with liver involvement	Limited predictive value; Lack of standardized cut-off values across different populations; Inconsistent results.
